# Choroidal Abnormalities in Pediatric NF1: A Cohort Natural History Study

**DOI:** 10.3390/cancers14061423

**Published:** 2022-03-10

**Authors:** Eleonora Cosmo, Luisa Frizziero, Giacomo Miglionico, Chiara Sofia De Biasi, Marisa Bruno, Eva Trevisson, Ilaria Gabbiato, Giulia Midena, Raffaele Parrozzani

**Affiliations:** 1 Department of Ophthalmology, University of Padova, 35128 Padova, Italy; eleonora.cosmo@aopd.veneto.it (E.C.); luisa.frizziero@unipd.it (L.F.); giacomo.miglionico@aopd.veneto.it (G.M.); chiarasofia.debiasi@aopd.veneto.it (C.S.D.B.); 2 IRCCS—Fondazione Bietti, 00198 Rome, Italy; marisa.bruno@fondazionebietti.it (M.B.); giulia.midena@fondazionebietti.it (G.M.); 3 Clinical Genetics Unit, Department of Women’s and Children’s Health, University of Padova, 35128 Padova, Italy; eva.trevisson@unipd.it (E.T.); ilaria.gabbiato@aopd.veneto.it (I.G.)

**Keywords:** neurofibromatosis, NF1, choroidal abnormalities

## Abstract

**Simple Summary:**

Choroidal abnormalities (CAs) have recently been introduced as one of the criteria for the diagnosis of neurofibromatosis type 1 (NF1). The aim of the present study was to assess the natural history of CAs in a large pediatric population affected by NF1, evaluating, on a long-term follow-up, CAs progression both in number and dimensions. To avoid bias due to the growing process of the eye, the CAs dimensions were normalized for the optic disc size. Our study demonstrated, in 99 eyes of 53 pediatric patients, an increase in the number, area and perimeter of CAs. The present study thus provides evidence that, in NF1 pediatric patients, CAs change with time, increasing both in number and dimensions, independently from the physiological growth of the eye. While the increase of the CAs number occurs particularly at an earlier age, the increase in the CAs dimensions is a slow process that remains constant during childhood.

**Abstract:**

The purpose of this study was to assess the long-term natural history of choroidal abnormalities (CAs) in a large pediatric neurofibromatosis type 1 (NF1) population, quantifying their progression in number and dimensions. Pediatric patients (<16 years old) affected by NF1 with a minimum follow-up of 3 years with at least one CA in one eye were consecutively recruited. Near-infrared (NIR) imaging was performed to identify CAs, which were quantified in number and size. The CAs area and perimeter were normalized for the optic disc dimensions to avoid possible bias related to the growing process of the eye. Ninety-nine eyes of 53 patients were evaluated. The CAs number, area and perimeter significantly increased during follow-up (*p* < 0.0001 for each parameter). The patient age at baseline was inversely correlated with the CAs number over time (coefficient = −0.1313, *p* = 0.0068), while no correlation was found between the patient age and CAs progression in size. In conclusion, we provide evidence that, in NF1 pediatric patients, CAs change over time, increasing both in number and dimensions, independently from the physiological growth of the eye. While the increase of the CAs number occurs particularly at an earlier age, the increase in the CAs dimensions is a slow process that remains constant during childhood.

## 1. Introduction

Neurofibromatosis type 1 (NF1) is a genetic disease affecting approximately 1 in 2500–3000 individuals [1,2,3]. It is characterized by a dominant autosomal transmission, with complete penetrance reached in 95% of cases by the age of 8, and it has a highly variable expression even in individuals of the same family [4,5].

Diagnosis can be made if two or more of the following criteria are present (one or more if a parent is affected): at least six café-au-lait macules (CALMs), axillary or inguinal freckling, at least two neurofibromas of any type or one plexiform neurofibroma, optic pathway glioma (OPG), at least two Lisch nodules (LNs) or two choroidal abnormalities (CAs) and distinctive osseous lesions [6]. The aforementioned criteria have been derived from a recent revision by the International Consensus Group on Neurofibromatosis Diagnostic Criteria (I-NF-DC), which introduced CAs as an ophthalmologic criterion because of its high specificity and sensitivity for the diagnosis of NF1 [6,7,8,9,10,11,12].

Historically, choroidal involvement in NF1 was considered a rare finding, mainly described in pathologic specimens as ovoid bodies into the choroid, consisting of hyperplastic Schwann cells, melanocytes and ganglion cells [13,14,15,16]. The in vivo detection of NF1-related choroidal abnormalities, which are completely asymptomatic and undetectable by conventional ophthalmoscopy or fluorescein angiography, was initially possible by means of indocyanine-green angiography [17] and, more recently, by near-infrared (NIR) reflectance imaging, a fully noninvasive tool that reveals CAs as bright patchy lesions, most often observed within the major vascular retinal arcades [18,19,20,21].

It has already been described that CAs tend to increase with patient age, both in the general NF1-population [19,22,23] and in the pediatric NF1-population [24], but none of the previous studies analyzed the same population longitudinally.

The aim of our study was to assess the natural evolution of CAs in a large cohort of NF1-affected children during a long-term follow-up, through an analysis that reduced the influence of the physiological growth of the eye.

## 2. Materials and Methods

This was an observational, longitudinal study performed in accordance with the tenets of the Declaration of Helsinki and approved by the Institutional Review Board. Informed consent was obtained from the legal guardian of each enrolled infant.

We retrospectively recruited pediatric patients affected by NF1 according to the revised diagnostic criteria for Neurofibromatosis type 1 of the International Consensus Group on Neurofibromatosis Diagnostic Criteria (I-NF-DC) [6], followed for surveillance in our Neurofibromatosis Eye Clinic between February 2012 and January 2021. Inclusion criteria were pediatric patients affected by NF1 [6], younger than 16 years old at their first clinical examination, with a follow-up of at least three years, who presented at least one CA in one eye. Exclusion criteria were a history of any ophthalmologic disease that may have affected the choroid and/or retina (e.g., uveitis, retinopathy of prematurity, maculopathy, congenital ocular malformations) or impaired adequate fundus visualization (e.g., congenital cataract or other media opacities). Eyes presenting optic disc abnormalities at baseline or developing optic disc oedema during follow-up (determining an inaccurate tracing of the optic disc margin that may affect the process of normalization of the CAs for the optic disc dimensions) were also excluded from the study.

At each evaluation, all patients underwent a complete ophthalmologic examination including: visual acuity assessment using age-appropriate visual function tests [25,26], slit lamp biomicroscopic examination and fundus examination using indirect ophthalmoscopy. The NIR reflectance modality of the Spectralis HRA + OCT (Heidelberg Engineering, Heidelberg, Germany) was used to evaluate the presence of CAs for each subject at each examination, as previously reported [8]. Briefly, images of the posterior pole and retinal midperiphery were captured using a 50° lens centered onto the posterior pole, with the help of an internal or external target (depending on age and patient cooperation) to maintain adequate fixation. The Spectralis automatic real time (ART) modality (16–100 averaged images) was used to avoid motion artifacts. The image resolution was 768 × 768 pixels.

**Figure 1:**
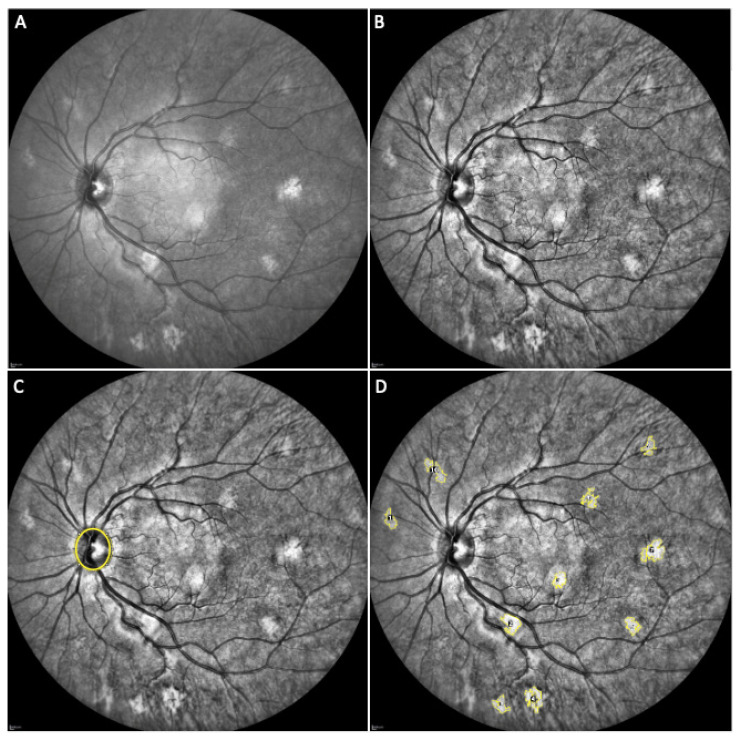
Elaboration of a near-infrared (NIR) image of a neurofibromatosis type 1 (NF1) patient eye with choroidal abnormalities (CAs). (**A**) original NIR image; (**B**) application of the “Enhance Local Contrast” tool with software standard parameters; (**C**) identification of the optic disc margin with the “Oval” tool; (**D**) identification of all the visible CAs, tracing of their margin with the “Wand Tracing” tool and assignment of a reference number.

The best quality image (which means the one with the best focus and resolution) was chosen to undergo a further analysis using the open-source available ImageJ software (National Institutes of Health, Bethesda, MD, USA). Each image was elaborated through the following steps (Figure 1): (a) the “Enhance Local Contrast” tool with software standard parameters was applied; (b) the “*Oval*” tool was used to manually identify the optic disc margin; three consecutive acquisitions were used to obtain the mean optic disc area (ODA) and the mean optic disc perimeter (ODP), and no significant variability was found among the measurements; (c) the “Wand Tracing” tool was used to automatically detect and trace margins of each visible CA; the area and the perimeter of each CA were automatically calculated. A reference number was assigned to each CA in each analyzed eye to allow comparison of the same lesion at each follow-up examination.

The area of CAs was expressed in pixel^2^ and then normalized for the ODA, and the perimeter of the CAs was expressed in pixel and then normalized for the ODC (to avoid possible bias related to the growing process of the eye). Thus, we obtained two measures for each CA, after normalization for optic disc dimensions: (a) CA area expressed in optic disc area (ODA); (b) CA perimeter expressed in optic disc perimeter (ODP). Data regarding the number, the area and the perimeter of CAs were collected at the baseline evaluation and at 1-year, 3-years, 5-years and 7-years follow-up visits.

We also collected data regarding NF1-related signs other than CAs that were present in each patient, such as the presence of six or more CALMs, axillary or inguinal freckling, two or more neurofibromas of any type or one plexiform neurofibroma, OPG, two or more LNs and distinctive osseous lesions.

Finally, the genetic mutation responsible for the disease was registered when available; thus, patients were divided into five groups according to the type of their mutation: missense, frameshift, splicing, deletions and nonsense, according to previously published methods of classification [10,27,28].

### Statistical Analysis

All study parameters were summarized according to usual methods of descriptive statistics for quantitative variables: the mean value and standard deviation were computed for the number, area, and perimeter of CAs.

In each eye, the total area of CAs (sum of the area of all CAs) and the total perimeter of CAs (sum of the perimeter of all CAs) were computed.

In this analysis, the number of CAs, normalized CAs area measure and normalized CAs perimeter measure were considered. Normalized measures were obtained by dividing the CAs area and perimeter by the optic disc’s area and perimeter respectively, measured at the same time.

Variation of the three measures across time was analyzed by means of a multiple linear regression model including not only time, but also the patient’s age and baseline value of the measure. The regression coefficient of the independent variable “Time” represented the rate of change (increase if positive, decrease if negative); the statistical inference of this coefficient gave us suggestions about the significance of correlation with time.

The comparisons of measures at 1, 3, 5, and 7 years compared to the baseline were analyzed by means of an ANOVA model adjusted for the patient’s age and baseline value of the dependent variable, followed by Tukey–Kramer post-hoc tests for multiple comparisons. The models were also adjusted for the replication of measures in both eyes of the same patient.

The correlation between CAs number at baseline and the number of NF-related signs presented at the age of 8 (the age at which NF1 has its maximal genetic penetrance) and type of genetic mutation were analyzed by means of a linear regression model adjusted for patient age.

For all analyses, SAS^®^ v. 9.4 statistical software was used (SAS Institute, Cary, NC, USA). Statistical results were interpreted as significant if *p* < 0.05.

## 3. Results

This study enrolled 99 eyes of 53 patients (31 males and 22 females), with a mean age of 7.1 ± 3.8 years old. Seven eyes that developed swelling of the optic nerve due to the presence of OPG during the follow-up were excluded from the analysis. All 99 eyes were examined at baseline and at least at 3-years follow-up (T3); 1-year follow-up (T1) was available for 80 eyes, 5-years follow-up (T5) for 59 eyes and 7-years follow-up (T7) for 36 eyes. The mean time duration of follow-up and the mean age at the last ophthalmologic evaluation were 5 ± 1.71 years and 7.46 ± 3.72 years, respectively. The child’s age at baseline did not relate to the follow-up length (*p* = 0.5981) during the study.

Data regarding the analysis on CAs number and dimensions are summarized in Table 1 and Table 2.

**Figure 2:**
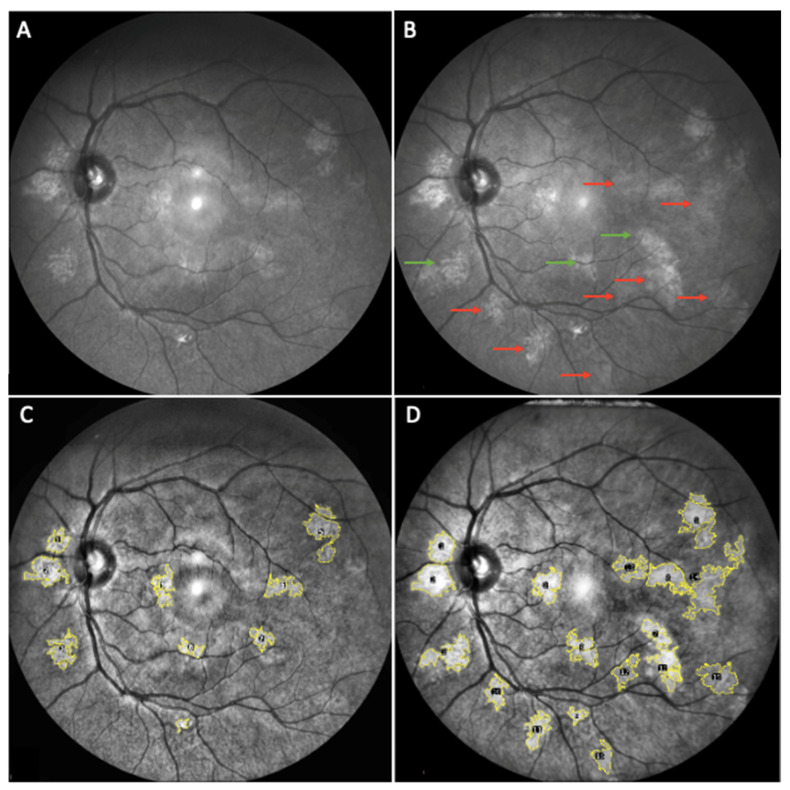
Evidence of the increased number and size of CAs in a NF1 patient. (**A**) (original NIR image) and (**C**) (elaborated image) represent the baseline evaluation; (**B**) (original NIR image) and (**D**) (elaborated image) represent the 3-years follow-up. In (**B**), red arrows indicate new CAs detected; green arrows indicate enlargement of previously observed CAs.

**Figure 3:**
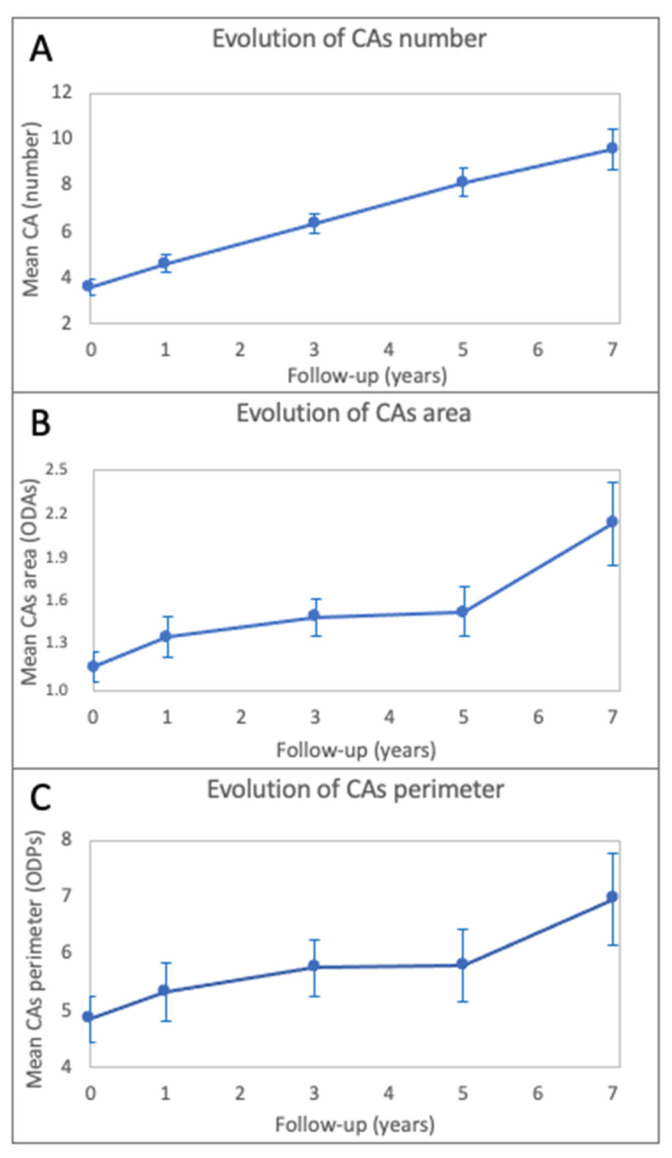
Graphical representation of the evolution along time of the CAs number (**A**), CAs area (**B**) and CAs perimeter (**C**); mean point and error bars are presented in the graphs.

The mean number of CAs increased from baseline (3.6 ± 3.2) to different follow-up visits (4.6 ± 3.5 at T1, 6.4 ± 4.1 at T3, 8.1 ± 4.8 at T5 and 9.6 ± 5.3 at T7), with a statistically significant trend (*p* < 0.0001) and an estimated growth rate of 0.82 CAs per year, adjusted for age and for the CAs number at baseline (Figure 2 and Figure 3).

The statistical significance was maintained even when considering separately intervals between different follow-up visits (*p* = 0.0418 for T5 vs. T7, *p* < 0.0001 for all the other time intervals): data are shown in Table 3.

The CAs number over time was negatively correlated with the patient’s age at baseline (coefficient = −0.1313; *p* = 0.0068) and positively correlated with the CAs number at baseline (coefficient = 1.1707; *p* < 0.0001).

Regarding dimensions, the mean area of CAs was 1.159 ± 0.975 ODAs at baseline, 1.367 ± 1.178 ODAs at T1, 1.495 ± 1.162 ODAs at T3, 1.534 ± 1.213 ODAs at T5 and 2.130 ± 1.564 ODAs at T7. The mean perimeter of CAs was 4.858 ± 3.745 ODPs at baseline, 5.320 ± 4.417 ODPs at T1, 5.753 ± 4.652 ODPs at T3, 5.803 ± 4.647 ODPs at T5 and 6.956 ± 4.536 ODPs at T7. Both the CAs area and perimeter increased significantly over time (*p* < 0.0001 for each parameter), adjusted for age and the CAs dimension at baseline, and the estimated growth rate per year was 0.10 ODA and 0.21 ODP, respectively (Figure 2 and Figure 3).

There was a positive statistically significant correlation between the CAs area and perimeter over time and the CAs dimensions at baseline (*p* < 0.0001 for both parameters), while no significant correlation was found with age (*p* = 0.8446 for area; *p* = 0.1736 for perimeter).

Considering the intervals between different follow-up visits, the increase in the CAs area was always significant, except for the interval between T1 and T3 and between T3 and T5; the increase in the CAs perimeter resulted significant only for the interval between baseline and T1, T3, T5 and T7 (Table 3).

We searched for a possible correlation between the CAs number at baseline (adjusted for age) and the severity of the disease expressed as quantity of NF-related signs (i.e., CALMs, atypical freckling, neurofibromas, OPG, LNs and distinctive osseous lesions), presented by each child at the age of 8, when penetrance of the disease in complete in 95% of cases [29]. The presence/absence of LNs was collected for all the studied subjects, while data regarding the other manifestations were available for 50 patients, since 3 children were followed for systemic features of the disease in other centers. The frequencies of other NF1-related signs are listed in Table 4. No significant correlation was found (*p* = 0.2746).

Finally, data collected on the genotype of the studied population are reported in Table 5. Five subjects were excluded from this analysis: a genetic test had not been performed for 2, had been performed in other centers and was not available for 2 and had not revealed pathogenic variants for 1. Complete details of the specific genotype associated with the CAs parameters of the study population are reported in Table S1 in the Supplementary Materials.

No significant correlation was found between the CAs number at baseline, adjusted for age, and the type of genetic mutation (i.e., missense, frameshift, splicing, deletions and nonsense) responsible for NF1 (*p* = 0.4204).

## 4. Discussion

Our study assessed the natural history of CAs in a large cohort of pediatric patients affected by NF1, regarding both the CAs number and dimensions.

A positive correlation between the CAs number and patient age has already been described [19,22,23,24]; nevertheless, the majority of previous studies were cross-sectional, thus providing only indirect evidence for the effect of time. We found a significant increase of the CAs mean number over time, with an estimated growth rate of 0.82 CAs per year (*p* < 0.0001). Therefore, our findings confirm on a longitudinal perspective what has been previously suggested by cross-sectional studies.

An inverse correlation was found between the CAs number and patient’s age at baseline, which means that a greater increase over time was seen in patients younger at baseline. To date, no reports on CAs age-specific incidence have been published, and by means of this inverse correlation, we can infer that CAs increase in number at an earlier age. This could facilitate their detection and allow clinicians to reach a diagnosis of NF1 in suspected children at an early age when the clinical picture seems incomplete.

Our study revealed that the increase in the CAs number was positively correlated with the CAs number at baseline, highlighting that the increase is greater in children that have more CAs at the baseline evaluation. We also found a positive correlation between the CAs size progression over time and the CAs baseline dimensions, meaning that subjects presenting wider CAs at their first evaluation were those who had a greater increase in their CAs size during follow-up. We evaluated if the CAs number at baseline could possibly be correlated with the quantity of other NF1-related signs (i.e., CALMs, atypical freckling, neurofibromas, OPG, LNs and distinctive osseous lesions) expressed by the children at the age of 8, when penetrance of the disease is complete in 95% of cases, or with the type of genetic mutation (i.e., missense, frameshift, splicing, deletions and nonsense) responsible for NF1. None of the two analyses showed a significant correlation. Thus, we can conclude that CAs are independent from the quantity of other NF1 present signs as well as from a specific NF1 genotype. Our findings agree with those reported by Cassiman et al., who did not find any significant difference regarding the presence of CAs comparing two groups of NF1 patients, one with truncanting and one with non-truncanting mutations [10].

Two recent papers described the evolution of CAs in terms of dimensions. Chilibeck et al. reported a progression in the number and dimensions of CAs in 26 eyes from 14 children [30], and Touze et al. showed an increase of CAs and of their size longitudinally analyzing a pediatric NF1-population [31]. However, none of these works considered the physiological increase in eyeball dimension during childhood that could bias the CAs surface increase. For this reason, we analyzed the CAs dimensions by means of a software that could enhance their distinctive brightness to better define and trace their boundaries and to normalize the obtained measures for the optic disc area and perimeter. Our study demonstrated a significant increasing trend in the CAs dimensions, with an estimated growth rate of 0.10 ODAs and 0.21 ODPs per year.

We did not find any significant correlation between the CAs size progression and patient age, suggesting a constant increase in the CAs dimensions, at least during childhood. This finding is in contrast with the report of Touzè et al. [31], who described that the slope of progression was maximal between the age of 8 and 12 years. Such a spike could be explained by a growth of the eye corresponding to the puberty period [32,33,34], whereas in our study, adjusting the CAs measures for the optic disc area and perimeter removed all the influence of the physiological growth of the eye. A possible limit of this study could have been the use of specific follow-up timepoints (irrespective of age), rather than age-specific time points. We decided on this approach to correlate Sa specific time period to the CAs modification. Nevertheless, to reduce the risk of an age-related bias, the statistical correction by the patient’s age at baseline was performed. A second potential limitation was that we did not search for a possible correlation between the CAs number and each NF1-related manifestation. Different from most of the other published works, our study was a longitudinal one, aiming to evaluate the quantity and size of CAs over time. The correlation between this quantitative changing parameter and a qualitative parameter (represented by each of the other NF-related signs) caught at a precise time-point would lack, in our opinion, statistical and meaningful strength. Moreover, the fact that all included subjects were characterized by the presence of CAs (as an inclusion criterion) may represent a remarkable bias in correlating this sign with the other NF-related manifestations. We did not include in our study design the assessment of the CAs thickness by means of OCT scans, mainly because CAs appear on OCT as irregular hyperreflective or hyporeflective foci in the choroid; thus, this technique is useful in confirming the choroidal localization of the abnormalities but it would not be precise in defining the real thickness of CAs, since their melanin component acts by blocking the underlying signal, giving a back-shadowing effect [21]. Anyway, it could be an interesting point to develop in the future. Moreover, a further evaluation of the behaviour of CAs in adulthood would also be interesting to investigate if a growth of the same persists even later in life.

## 5. Conclusions

In conclusion, this is the first report assessing longitudinally, on a long-term basis, the natural evolution of CAs in an NF1 pediatric population considering and eliminating the influence of eye growth. We demonstrated that the CAs number increases particularly at an early age, while a slow growth in size is significant and constant during childhood.

## Figures and Tables

**Table 1:** Evolution along time of CAs number and size.

CAs Parameter	Baseline	T1	T3	T5	T7
	*n* = 99	*n* = 80	*n* = 99	*n* = 59	*n* = 36
Number (mean ± sd)	3.6 ± 3.2	4.6 ± 3.5	6.4 ± 4.1	8.1 ± 4.8	9.6 ± 5.3
Area ^1^ (mean ± sd)	1.159 ± 0.975	1.367 ± 1.178	1.495 ± 1.162	1.534 ± 1.213	2.130 ± 1.564
Perimeter ^2^ (mean ± sd)	4.858 ± 3.745	5.320 ± 4.417	5.753 ± 4.652	5.803 ± 4.647	6.956 ± 4.536

^1^ CAs areas are expressed in ODA; ^2^ CAs perimeters are expressed in ODP.

**Table 2:** Coefficients and *p*-values of the multiple linear regression analysis conducted on the variation of the CAs number, area and perimeter over time; the model also included the patient’s age and baseline value of the measure; statistically significative values are in bold.

Parameter	CAs Number	CAs Area	CAs Perimeter
Time	0.8213, ***p* < 0.0001**	0.1047, ***p* < 0.0001**	0.2102, ***p* < 0.0001**
Age	−0.1313, ***p* = 0.0068**	−0.0019, *p* = 0.8446	0.0514, *p* = 0.1736
Baseline value	1.1707, ***p* < 0.0001**	1.1274, ***p* < 0.0001**	1.1012, ***p* < 0.0001**

**Table 3:** *p*-values and coefficients of the multiple linear regression analysis conducted on the variation of the CAs number, area and perimeter over time, considering intervals between different follow-up visits; statistically significative values are in bold; the patient’s age at baseline and baseline value of the measures were the covariates included in the models.

Time Interval	CAs Number		CAs Area		CAs Perimeter	
	*p*-Value	Coefficient	*p*-Value	Coefficient	*p*-Value	Coefficient
Baseline vs. T1	**<0.0001**	−1.1721	**0.0004**	−0.2427	**0.0126**	−0.7167
Baseline vs. T3	**<0.0001**	−2.7677	**<0.0001**	−0.3362	**0.0003**	−0.8943
Baseline vs. T5	**<0.0001**	−4.4944	**<0.0001**	−0.4637	**<0.0001**	−1.2635
Baseline vs. T7	**<0.0001**	−5.5130	**<0.0001**	−0.8906	**<0.0001**	−1.5382
T1 vs. T3	**<0.0001**	−1.5956	0.5015	−0.0935	0.9316	−0.1776
T1 vs. T5	**<0.0001**	−3.3223	**0.0113**	−0.2210	0.2190	−0.5468
T1 vs. T7	**<0.0001**	−4.3409	**<0.0001**	−0.6479	0.0697	−0.8215
T3 vs. T5	**<0.0001**	−1.7268	0.2968	−0.1275	0.5781	−0.3692
T3 vs. T7	**<0.0001**	−2.7453	**<0.0001**	−0.5545	0.2204	−0.6439
T5 vs. T7	**0.0418**	−1.0185	**<0.0001**	−0.4270	0.9186	−0.2747

**Table 4:** Frequencies of other NF1-related signs presented by children at the age of 8 years old.

NF-Related Sign	Patients Number	Frequency	
		*n*	*%*
Lisch nodules (at least 2)	53	38	71.7%
Café-au-lait macules	50	50	100%
Atypical freckling	50	50	100%
Neurofibromas (at least 2 or 1 plexiform)	50	27	54%
Optic pathway glioma	50	18	34.62%
Distinctive osseous lesions	50	5	10%

**Table 5:** Frequencies of different types of genetic mutations responsible for the disease, collected in 48 patients.

Type of Mutation	Frequency	
	*n*	*%*
Missense	8	16.7%
Frameshift	16	33.3%
Splicing	7	14.6%
Deletion	3	6.2%
Nonsense	14	29.2%
Total	48	100%

## Data Availability

The data presented in this study are available in the Article. Eventual additional data are available on request from the corresponding author.

## Supplementary Materials

The following supporting information can be downloaded at: https://www.mdpi.com/article/10.3390/cancers14061423/s1, Table S1: Details of study population genotype and associated CAs parameters.
