# Supplementary material for: Choroidal Abnormalities in Pediatric NF1: A Cohort Natural History Study
Source: Cancers (Basel). 2022 Mar 10;14(6):1423. doi: 10.3390/cancers14061423 (PMC8946817; doi:10.3390/cancers14061423)
Supplement: Supplementary file 1 [file cancers-14-01423-s001.zip › cancers-1624867-supplementary.pdf]

Table S1. Details of study population genotype and associated CAs parameters (number, area and perimeter). Patients sex, age at baseline and duration of follow-up are also reported.

Patient	Sex [1]	Age at Baseline (y.o.)	Mutation	Type of Mutation	CAs Number (Sum of Both Eyes)		CAs area [2] (Sum of Both Eyes)		CAs Perimeter [3] (Sum of Both Eyes)		Follow-up Duration
					min	max	min	max	min	max	
1	M	3	Genetic test not performed								
2	M	7.4	NF1 gene microdelection	delection	2	4	0.34	0.42	1.16	1.42	3 years
3	F	7.4	NM_000267.3: c.3457_3560del p.(Leu1153Metfs *4)	frameshift	12	24	4.82	6.66	14.38	24.14	7 years
4	F	3.5	NM_000267.3: c.7394+2delT	splicing	1	3	0.51	0.66	2.62	5.15	3 years
5	M	15.5	NM_000267.3: c.586+2T>A	splicing	14	25	1.16	2.23	7.95	12.24	7 years
6	F	4.6	NM_000267.3: c.4871T>G p.(Leu1624Arg)	missense	3	13	1.84	2.92	6.76	8.70	7 years
7	M	13.8	NM_000267.3: c.3525_3526delAA p.(Arg176Serfs *18)	frameshift	15	33	3.62	5.46	15.97	21.93	7 years
8	M	13.4	delection of all the exons of NF1 gene	delection	9	11	2.92	4.37	11.66	13.46	5 years
9	M	4.3	NM_000267.3: c.2088G>A p.(Trp696 *)	nonsense	6	22	2.33	3.09	8.46	14.59	5 years
10	M	6.4	Genetic test didn’t reveal pathogenic variants								
11	M	9.3	NM_000267.3: c.662G>A p.(Trp221 *)	nonsense	2	4	0.32	0.69	1.62	2.06	7 years
12	F	3.1	NM_000267.3: c.998delA p.(Tyr333Serfs *42)	frameshift	1	1	0.44	0.54	2.99	4.66	3 years
13	F	8.5	Genetic test not available								
14	M	4.4	NM_000267.3: c.4083delT p.(Arg1362Glufs*23)	frameshift	2	2	0.58	0.67	3.19	4.88	3 years
15	M	4.1	NM_000267.3: c.7267dup p.(Thr2423Asnfs*4)	frameshift	1	13	0.56	2.38	2.97	6.88	7 years
16	F	4.5	NM_000267.3: c.2531T>C p.(Leu844Pro)	missense	6	18	1.55	2.47	6.24	11.24	5 years
17	F	3.3	Genetic test not performed								

18	F	5.8	NM_000267.3: c.1466A>G r.1464_1525del62nt p.(Ser488Argfs *1)	frameshift	1	2	0.54	0.92	3.08	5.83	5 years
19	M	7.5	NM_000267.3: c.2851-6_2851-3delCTTT p.(Leu952fs)	frameshift	4	11	1.23	1.53	5.14	7.04	5 years
20	F	14.8	NM_000267.3: c.7619C>A p.(Ser2540 *)	nonsense	14	16	5.80	6.66	23.02	25.21	3 years
21	M	3.2	NM_000267.3: c.2617C>G p.(Arg873Gly)	missense	1	5	0.43	0.51	2.09	2.64	3 years
22	M	7.7	NM_000267.3: c.889-2A>G	splicing	7	15	2.51	2.69	10.84	14.31	3 years
23	M	4.1	NM_000267.3: c.910C>T p.(Arg304 *)	nonsense	4	17	1.81	2.24	7.03	8.19	3 years
24	M	4.1	NM_000267.3: c.3911T>G p.(Leu1304 *)	nonsense	1	3	0.55	1.64	3.41	5.54	5 years
25	M	9.6	NM_000267.3: c.3301C>T p.(Gln1101 *)	nonsense	22	42	6.04	9.48	22.53	31.26	7 years
26	F	5	NM_000267.3: c.6942dupC p.(Ala2315Argfs *4)	frameshift	1	5	1.12	1.75	2.97	5.63	5 years
27	M	6.8	NM_000267.3: c.2990+1G>A r.2851_2990del140	splicing	11	29	2.28	3.40	10.63	20.55	5 years
28	F	4.4	NM_000267.3: c.574C>T p.(Arg192 *)	nonsense	11	29	2.94	4.61	11.08	16.31	7 years
29	M	6.2	NM_000267.3: c.7125delA p.(Tyr2377Thrfs *20)	frameshift	6	19	3.60	6.50	11.04	14.05	7 years
30	F	4.3	NM_000267.3: c.7901_7902insTGTTG p.(His2637Leufs *23)	frameshift	3	5	0.46	0.72	2.06	2.63	3 years
31	M	10.2	NM_000267.3: c.7882dupG p.(Val2628Glyfs *11)	frameshift	15	24	3.18	9.45	15.85	25.26	7 years
32	M	5.5	NM_000267.3: c.1858delA p.(Ser620Valfs *11)	frameshift	2	6	0.30	1.17	1.55	3.10	3 years
33	F	4.1	NM_000267.3: c.4172G>C p.(Arg1391Thr)	missense	13	25	4.45	7.28	20.35	27.19	7 years
34	F	3.4	NM_000267.3: c.2252-2A>G	splicing	7	18	1.32	2.41	5.92	12.77	3 years
35	F	2.8	NM_000267.3: c.3163C>T p.(Gln1055 *)	nonsense	1	4	0.39	0.58	2.11	2.26	3 years

36	F	5.9	Genetic test not available									
37	F	5.6	NM_000267.3: c.4267A>G p.(Lys1423Glu)	missense	13	28	4.89	8.21	18.49	25.93	3 years
38	M	4.4	NM_000267.3: c.7331delA p.(Asp2444Valfs *24)	frameshift	1	1	0.26	0.77	2.11	5.68	5 years
39	F	13.9	NM_000267.3: c.6709C>T p.(Arg2237 *)	nonsense	6	11	0.71	2.76	4.55	8.84	7 years
40	M	4.5	NM_000267.3: c.2530C>T p.(Leu844Phe)	missense	4	16	1.47	2.22	7.45	11.82	7 years
41	M	11.3	NM_000267.3: c.6709C>T p.(Arg2237*)	nonsense	4	10	2.44	2.68	9.19	10.99	3 years
42	M	6.2	NF1 gene microdelection	delection	1	3	0.61	1.13	3.63	8.42	7 years
43	M	5.7	NM_000267.3: c.6724C>T p.(Gln2242*)	nonsense	2	11	2.28	2.89	2.53	3.40	5 years
44	M	9.4	NM_000267.3: c.3457_3460delCTCA p.(Leu1153Metfs*4)	frameshift	4	6	1.13	1.24	4.66	4.98	3 years
45	F	8.7	NM_000267.3: c.4108C>T p.(Gln1370*)	nonsense	17	21	3.94	5.16	16.78	26.17	3 years
46	M	6.4	NM_000267.3: c.1458_1459delAA p.(Arg487Lysfs *3)	frameshift	12	28	3.03	4.12	11.39	14.42	5 years
47	F	13.6	NM_000267.3: c.3916C>T p.(Arg1306 *)	nonsense	19	31	2.21	3.12	14.15	18.17	5 years
48	M	3.7	NM_000267.3:c.1756_1759delACTA p.(Thr586Valfs *18)	frameshift	2	12	0.60	1.20	2.40	3.82	5 years
49	M	15.8	NM_000267.3: c.3327A>T p.(Leu1109Phe)	missense	14	14	3.09	4.56	10.44	12.62	3 years
50	M	15.4	NM_000267.3: c.1885G>A p.(Gln629Arg)	missense	19	20	7.02	9.76	26.49	37.43	5 years
51	F	6.2	NM_000267.3: c.5205+1G>C	splicing	1	11	0.41	0.65	1.81	3.41	7 years
52	M	4	NM_000267.3: c.7806+1G>T r.7676_7806de1131	splicing	3	5	0.47	0.96	2.53	3.66	7 years
53	F	8.2	NM_000267.3: c.6709C>T p.(Arg2237 *)	nonsense	15	21	5.39	7.47	21.17	28.69	3 years

[1] M= male; F= female. [2] CAs areas are expressed in ODA. [3] CAs perimeters are expressed in ODP.
